# Knowledge of cervical cancer risk factors and symptoms among women in a refugee settlement: a cross-sectional study in northern Uganda

**DOI:** 10.1186/s13031-020-00328-3

**Published:** 2020-12-03

**Authors:** Winnie Adoch, Christopher Orach Garimoi, Suzanne E. Scott, Geoffrey Goddie Okeny, Jennifer Moodley, Henry Komakech, Fiona M. Walter, Amos Deogratius Mwaka

**Affiliations:** 1 School of Public Health, College of Health Sciences, Makerere University, P.O Box 7072, Kampala, Uganda; 2 Department of Community Health and Behavioural Sciences, School of Public Health, College of Health Sciences, Makerere University, P.O Box 7072, Kampala, Uganda; 3 Centre for Oral, Clinical & Translational Sciences, Faculty of Dentistry, Oral & Craniofacical Sciences, King’s College London, London, UK; 4 Women’s Health Research Unit, School of Public Health and Family Medicine, Faculty of Health Sciences, University of Cape Town, Anzio Road. Observatory, Cape Town, 7925 South Africa; 5 Cancer Research Initiative, Faculty of Health Sciences, University of Cape Town, Anzio Road, Observatory, Cape Town, 7925 South Africa; 6 The Primary Care Unit, Department of Public Health & Primary Care, University of Cambridge, Cambridge, UK; 7 Department of Medicine, School of Medicine, College of Health Sciences, Makerere University, P.O Box 7072, Kampala, Uganda

**Keywords:** Refugee settlement, Awareness, Cervical cancer, Risk factors, Symptoms

## Abstract

**Background:**

There are limited data on awareness of cervical cancer risk factors and symptoms among refugee populations living in Uganda. In this study, we sought to determine the awareness and knowledge of cervical cancer risk factors and symptoms among women in Palabek refugee settlement, northern Uganda.

**Methods:**

We conducted a cross-sectional study. 815 women (aged 18–60 years) were randomly selected using multistage sampling in Palabek refugee settlement. Data were collected using pre-tested, structured questionnaires. Logistic regression models were used to determine magnitudes of association between socio-demographic and health system factors, and knowledge on cervical cancer risk factors and symptoms.

**Results:**

The majority of participants (53%, *n* = 433) were young (18–29 years), married (68%, *n* = 553), and did not have formal employment (93%, *n* = 759). Less than half (40%, *n* = 325) had heard of cervical cancer. Of those who had heard, most recognized multiple male sexual partners, early onset of sexual intercourse and HPV infections as risk factors for cervical cancer (93%, *n* = 295; 89%, *n* = 283; and 86%, *n* = 271 respectively). Median knowledge score for risk factor recognition = 7 (IQR: 3–9). Median knowledge score for symptoms recognition = 7 (IQR: 1–10). Half of women (50%, *n* = 409) correctly recognized 7 to 11 symptoms of cervical cancer, with vaginal bleeding between menstrual periods, pelvic pain, and vaginal bleeding during/after sexual intercourse recognized by 58, 52 and 54% respectively. Single women (OR = 0.59 (95%CI: 0.38–0.94), and women that lived farther than 1 kilo meter from nearest health facility in South Sudan (OR = 0.36–0.49 (95%CI: 0.26–0.84) were less likely to be knowledgeable of symptoms of cervical cancer.

**Conclusion:**

A significant proportion of women in Palabek refugee settlement had not heard about cervical cancer. Refugee health services providers could increase awareness of cervical cancer risk factors and symptoms through health education in order to promote risk reduction behaviours and guide women during symptoms appraisal. Single women and those who lived more than one kilo metre from nearest health facility in home country could be a priority group for awareness intervention in the settlement.

## Background

Worldwide, there were 569,847 new cases of cervical cancer diagnosed in 2018; and there were 311,365 cancer deaths. 85% of the new cases and deaths from cervical cancer occurred in low- and middle-income countries (LMICs) [1]. In the high-income countries including the UK and USA, cervical cancer incidence and mortality rates have fallen during the past 30 years largely due to increased awareness, cervical screening, and prompt treatment of pre-cancerous lesions, as well as general improvement in the treatment of invasive cervical cancer [2, 3]. However, in Sub-Saharan Africa cervical cancer is still the second most common cause of cancer death among women [4, 5]. In Sub-Saharan Africa, 65–85% of cervical cancer patients are diagnosed at advanced stage and have poor treatment outcomes [6–9].

The incidence and mortality from cervical cancer in Sub-Sahara African refugee settings are likely higher than in the host population. Generally, refugees and people in internally displaced persons camps (IDPs) have poorer health and higher incidence of sexually transmiited infections including HIV and hepatitis B [10–13]. Refugee women experience greater episodes of sexual violence and forced marriages [14, 15]. Forced sex and sex for money with several different men are common in displaced populations [16] and potentially increase exposure of women in refugee settings to multiple strains of oncogenic HPV infections, thereby increasing their long term risk to development of invasive cervical cancer. In Lebanon, Syrian refugees were found to have poorer access to healthcare services than the host community [17]. Poor access to health information potentially undermines awareness of refugee women regarding cancer risk factors and symptoms. A study among 176 Syrian refugees in Greece revealed low awareness of cervical cancer risk factors and symptoms. Less than half of the women recognized post menaopausal bleeding (44.3%), and intermentsrual bleeding (34.1%) as symptoms of cervical cancer [18]. Another study that recruited more Syrian refugees (417) in Lebanon also showed low awareness of cancers among the refugees [19]. Similarly, African refugees in Australia demonstrated significantly lower level of awareness about cervical cancer compared to non-refugee African women [20]. There is therefore evidence that refugees, especially women and children tend to be at disadvantage regarding access and utilization of several essential services including cancer information and care. In the USA, the risk of reproductive health problems including breast and cervical cancers were significantly higher among recently settled refugee women compared to non-refugee women [21]. Poor access to healthcare services mainly because of low incomes, increased cost of basic services, lack of continuity of care if patients become displaced and limited follow up are some of the challenges to cancer prevention in refugee settings [22]. In Uganda, there is some evidence that in certain refugee settings, especially the rural settlements, access to primary care health services provided by some international humanitarian organizations including United Nations High Commission for Refugees (UNHCR) is better among refugees than the host communities [23]. However, access and utilization of health services are poorer for refugees in urban settlements than host communities [24, 25]. Understanding the interplay of social determinants of health and health system factors including access to specialized care are critical to appropriate planning for refugee health services in the refugee settlements.

There are several refugee settelments in Sub-Saharan Africa with very large populations of women and children [26]. For example, in Uganda there are an estimated 1.2 millon refugees [27]. In 2018, Uganda hosted an estimated 785,104 South Sudanese refugees. This figure was expected to rise to 861,590 by end of 2019. In Lamwo district, there were an estimated 53,780 South Sudanese refugees by end of 2019, more than 50% of whom were women and children [28, 29]. The burden of cervical cancer and the awareness of cervical cancer risk factors and symptoms are not adequately documented among these refugee women. This study assessed knowledge of cervical cancer risk factors and symptoms among a large population of refugee women in Palabek refugee settlement, northern Uganda, in order to inform targeted interventions on prevention, risk reduction behaviours and early detection of symptomatic cervical cancer among the refugee women.

## Methods

### Study setting

This study was conducted in Palabek refugee settlement, northern Uganda. Palabek refugee settlement is one of the newest refugee settlements in Uganda established in April 2017 [30]. It is divided into seven Zones; the zones are further subdivided into 35 blocks. Palabek refugee settlement has four health centres [31]. The settlement hosts an estimated 53,780 refugees [28, 29].

#### Study design and sampling procedure

This was a cross-sectional survey. We used a multilevel sampling approach to select the study participants. We obtained the updated list of zones and blocks within the settlement with their respective population sizes from the settlement commandant at the Office of the Prime Minister (OPM). This list was used as the sampling frame. The number of women selected from each zone was based on probability proportionate to sizes of the blocks. Two blocks were randomly selected from each of the 7 zones. The number of women selected per block was obtained by dividing the total population in each block with the total population of all the blocks in the zone then multiplied by the sample size calculated per zone. Systematic random sampling of every “10th” (interval) household was done until the required number of participants from each selected block was achieved. The beginning household was selected by simple random sampling of one out of 10 listed households in every selected blocks. We identified eligible households with the help of village health teams and the local leaders in the zones/blocks. Data collection was conducted during May 2019. In each household, one woman aged 18–60 years was selected by simple random sampling from a list of eligible women within the household. Potential particpants (20 households) who were never found home on two visits during field data collection were replaced. Women with self-reported or documented diagnosis of cervical cancer or those reported by family members and or judged by research assistants as not mentally sound or with speech impairments were to be excluded. 30 women declined to participate for various reasons. No woman fulfilled the exclusion criteria.

#### Data collection

A standardised pretested questionnaire adapted from the African Women Awareness of CANcer (AWACAN) tool (www.awacan.online) was used to collect data [32]. Data from the pretest was used to refine the questionnaire to make it relevant and suitable to the circumstance of this study setting. For example, the questions on assets were rephrased to refer to when they were still in South Sudan rather than in the settlement where most of them own items distributed equitably by humanitarian organizations. Piloting of the tool was done with 30 women from one of the blocks not selected for the main study. The Acoli version of the AWACAN tool was used in this study. The population in this settlement predominantly speak Acoli language. As part of quality control procedure, experienced Research Assistants (RAs) with bachelor degree level education were selected and trained for two days on the study tool, objectives of the study, consenting procedures, cervical cancer risk factors and symptoms, and provided a brief background on cervical cancer as a disease. WA supervised the research assistants during data collection. Each RA interviewed a participant independently. Data were collected using android phones loaded with the Open Data Kit (ODK) software.

#### Data management

At the end of each workday, WA uploaded data collected from the ODK into Epidata 3.1 and reviewed the data for completeness, accuracy and consistency. In seven instances, the RAs revisited the households where data were not complete and or aspects of the data not clear. The biostatistician (GOG) reviewed data from 10% of randomly selected participants to ensure data quality. There were no significant inconsistencies detected in data entry and capture. Knowledge of cervical cancer risk factors and symptoms was assessed based on scores developed from the list of risk factors and symptoms presented to the participants for recognition: *(i) Cervical cancer risk factors: 10 risk factors were assessed. A participant scored 1 or 0 if she responded “Yes” or “No” respectively to a known cervical cancer risk factor. The highest expected knowledge score for any one participant = 10, while the lowest score = 0. The calculated median knowledge score was 7. Participants with knowledge scores of 0–6 were considered to have low knowledge (less knowledgeable) while those with scores of 7 and above were considered to have high knowledge (highly knowledgeable). (ii) Cervical cancer symptoms: 11 symptoms were assessed. A participant scored 1 or 0 if she responded “Yes” or “No” respectively to a symptom known to be associated with cervical cancer. The highest expected knowledge score for any one participant = 11, while the lowest score = 0. The calculated median knowledge score was 7. Participants with knowledge scores of 0–6 were considered to have low knowledge (less knowledgeable) while those with scores of 7 and above were considered to have high knowledge (highly knowledgeable).*

### Data analysis

Univariate analysis was used to obtain the descriptive statistics (demographic characteristics) of participants. Measures on knowledge scores included median and interquartile ranges. Knowledge scores were categorized into binary outcome around the median scores as less knowledgable (below median) and highly knowledgeable (median and above). Bivariate analysis using Chi square tests were conducted to determine assocations between the binary knowledge scores on risk factors and symptoms of cervical cancer with socio-demographic correlates. Multivariate logistic regression models were applied on categorical variables with binary outcomes (split around the median scores) to determine the magnitudes of associations between knowledge of risk factors and symptoms of cervical cancer with selected independent variables. Statistical significance was set at *p*-value < 0.05. Odds ratios and accompanying 95% confidence intervals have been reported.

## Results

### Socio demographic characteristics of participants

More than half of participants (53%, *n* = 433) were young (18–29 years), and were married (68%, *n* = 553). Most participants were not formally employed (93%, *n* = 759). Less than half of the women (40%, *n* = 325) self-reported having heard of cervical cancer before this study (Table 1).

**Table 1:** Socio demographic characteristics of participants

Variables	Frequency, n	Percentage (%)
**Age (Years),** ***N*** **= 815**		
18–29	433	53.1
30–45	302	37.1
46–60	80	9.8
**Marital status, N = 815**		
Married/Living together	553	67.9
Separated/Divorced	60	7.4
Single	107	13.1
Widowed	95	11.6
**Level of education, N = 815**		
None	195	23.9
Primary	524	64.3
Secondary	88	10.8
Tertiary	8	0.9
**Tribe, N = 815**		
Acoli	785	96.3
Others	30	3.7
**Employment, N = 815**		
No	759	93.1
Yes	56	6.9
**Ever heard of cervical cancer before this study,** ***N*** **= 815**		
Yes	325	39.9
No	490	60.1
**Distance to the nearest health facility in South Sudan,** ***N*** **= 815**		
< 1 km	238	29.2
1–5 km	494	60.6
> 5 km	68	8.4
Do not know	15	1.8

### Recognition of cervical cancer risk factors

Knowledge of cervical cancer risk factors was assessed among the 325 women (40%) who self-reported they had heard of cervical cancer before this study. Recognition of cervical cancer risk factors was quite good; 93% (295/319) recognized that having multiple sexual partners (> 3 men) increases a woman’s risk of developing cervical cancer. Similarly, 87% (271/314) recognized infection with the human papilloma virus (HPV) as a risk factor for cervical cancer. However, less than half of participants recognized smoking cigarettes (38%, 113/300) as risk factor for cervical cancer (Table 2).

**Table 2:** Recognition of cervical cancer risk factors

	***Recognized as cervical cancer risk factor***	
***Risk factors assessed***	***Yes,*** N (%)	***No*** N (%)
*Having many sexual partners (greater than 3) in a lifetime.*	*295 (92.5)*	*24 (7.5)*
*Having sex at a young age (17 years or younger)*	*283 (88.5)*	*37 (11.6)*
*Being infected with other sexually transmitted diseases (other than HIV or Human Papillomavirus)*	276 (88.2)	37 (11.8)
*Getting a sexually transmitted infection called Human Papillomavirus (HPV)*	271 (86.3)	43 (13.7)
*Not going for regular screening /testing for cervical cancer*	*222 (69.8)*	*96 (30.2)*
*Having a sexual partner who is not circumcised*	*215 (67.8)*	*102 (32.2)*
*HIV/AIDS*	206 (66.7)	103 (33.3)
*Giving birth to three or more children*	*155 (49.8)*	*156 (50.2)*
*Smoking any cigarette at all*	*113 (37.7)*	*187 (62.3)*
*Using birth control, family planning for more than 5 years*	172 (31.1)	382 (68.9)
*Being bewitched/witchcraft*	*18 (6.4)*	*265 (93.6)*

### Cervical cancer risk factor knowledge scores

Half of the 325 participants who responded to the questions on the 10 cervical risk factors could recognize at least 6 risk factors correctly. Median number of risk factors recognized was 7 (IQR: 3–9). 11% could correctly recognize 7 risk factors, while 40% recognized 8–10 risk factors correctly (Table 3). However, there were no associations between sociodemographic variables including age, marital status and educational attainment with being knowledgeable about cervical cancer risk factors except for employment status (Table 5).

**Table 3:** Median knowledge scores for risk factors and symptoms of cervical cancer

Knowledge scores	Number	Percentage
**Risk factors for cervical cancer (N = 325)**		
Median score [7] (IQR: 3.0–9.0)	35	10.8
Below median score	160	49.2
Greater than median score	130	40.0
**Symptoms of cervical cancer (N = 815)**		
Median score [7] (IQR: 1.0–10.0)	39	4.8
Below median score	406	49.8
Greater than median score	370	45.4

*IQR* Interquartile range

### Recognition of cervical cancer symptoms

Recognition of cervical cancer symptoms were assessed for the individual symptoms among all the participants. Persistent lower abdominal pain was the most recognized (65%, *n* = 532) symptom of cervical cancer. Other symptoms recognized by more than half of participants included persistent smelly vaginal discharge (63%, *n* = 509)*,* vaginal bleeding after menopause (60%, *n* = 489) and vaginal bleeding between menstrual period**s** (58%, *n* = 469) (Table 4).

**Table 4:** Recognition of cervical cancer symptoms

***Symptoms read out to participants***	***Recognized as symptoms of cervical cancer***		
	***Yes*** ***N (%)***	***No*** ***N (%)***	***Don’t know*** ***N (%)***
*Persistent lower abdominal/pelvic pain*	*532 (65.3)*	*49 (6.0)*	*234 (28.7)*
*A persistent smelly vaginal discharge*	509 (62.5)	70 (8.6)	236 (28.9)
*Vaginal bleeding after menopause*	*489 (60.0)*	*64 (7.9)*	*262 (32.2)*
*Vaginal bleeding between menstrual period****s***	*469 (57.6)*	69 (8.5)	277 (33.9)
*Menstrual periods that are longer or heavier than usual*	*464 (56.9)*	*82 (10.1)*	*269 (33.0)*
*Persistent lower back pain*	459 (56.3)	95 (11.7)	261 (32.0)
*Vaginal bleeding during or after sex*	*437 (53.6)*	*95 (11.7)*	*283 (34.7)*
*Discomfort or pain during sex*	*424 (52.0)*	*94 (11.5)*	*297 (36.4)*
*Blood in urine/stool(faeces)*	*423 (51.9)*	*71 (8.7)*	*321 (39.4)*
*Persistent diarrhoea*	*254 (31.2)*	*152 (18.7)*	*409 (50.2)*
*Unexplained weight loss*	*197 (24.2)*	*120 (14.7)*	*498 (61.1)*

### Cervical cancer symptoms knowledge scores

Half of the 815 participants could correctly recognize at least 6 cervical cancer symptoms. Median number of symptoms recognized = 7 (IQR: 1–10). 5% could correctly recognize 7 cervical cancer symptoms, while 51.2% correctly recognized 7–11 cervical cancer symptoms (Table 3). Upon adjusting for other socio-demographic variables, single women (OR = 0.59 (95%CI: 0.38–0.94), and women that lived farther than 1 kilo meter from nearest health facility in South Sudan (OR = 0.36–0.49 (95%CI: 0.26–0.84) were less likely to be knowledgeable of symptoms of cervical cancer (Table 6).

### Factors associated with knowledge of cervical cancer risk factors

On adjusting for age, level of education, employment status, tribe, and distance to the nearest health facility back in South Sudan, single participants were 41% less likely to be knowledgeable about cervical cancer symptoms compared to participants who were married (OR = 0.59; 95%CI: 0.38–0.94). Similarly, on adjusting for participants’ sociodemographic charateristics, those who lived farther than 1 km from the nearest health facility back in South Sudan were 51–64% less likely to be knowledgeable of cervical cancer symptoms compared to participants who lived within 1 km (Table 5).

**Table 5:** Knowledge of cervical cancer risk factors and socio-demographic correlates

Socio-demographic variables	Knowledgeable on cervical cancer risk factors		Odds ratios (OR)	
	Yes N (%)	No N (%)	Crude OR (95% CI)	Adjusted OR^**b**^ (95% CI)
**Age (Years),** ***N*** **= 325**				
18–29	82 (49.7)	84 (52.5)	Ref	Ref
30–44	66 (40.0)	56 (35.0)	1.20 (0.76–1.93)	1.15 (0.70–1.87)
45–60	17(10.3)	20 (12.5)	0.87 (0.43–1.78)	0.89 (0.42–1.88)
**Marital status,** ***N*** **= 325**				
Married/Living together	109 (66.1)	117 (73.1)	Ref	Ref
Separated/Divorced	23 (13.9)	11 (6.9)	**2.24 (1.05–4.82)**	2.06 (0.94–4.53)
Single	15 (9.1)	14 (8.7)	1.15 (0.53–2.17)	1.31 (0.59–2.96)
Widowed	18 (10.9)	18 (11.3)	1.07 (0.72–1.21)	1.04 (0.48–2.28)
**Level of education,** ***N*** **= 325**				
None	29 (17.6)	30 (18.8)	Ref	Ref
Primary	116 (70.3)	108 (67.5)	1.11 (0.62–1.97)	1.13 (0.60–2.12)
Secondary	17 (10.3)	21 (13.1)	0.84 (0.37–1.90)	0.83 (0.34–2.04)
Tertiary	3 (1.8)	1 (0.6)	3.10 (0.30–31.58)	2.10 (0.19–23.19)
**Tribe,** ***N*** **= 325**				
Acoli	161 (97.6)	156 (97.5)	Ref	Ref
Others	4 (2.4)	4 (2.5)	0.97 (0.24–3.94)	1.09 (0.26–4.55)
**Employment,** ***N*** **= 325**				
No	132 (80.0)	147 (91.9)	Ref	Ref
Yes	33 (20.0)	13 (8.1)	**2.82 (1.43–5.60)**	**2.77 (1.36–5.62)**
**Distance to the nearest HF in Sudan,** ***N*** **= 325**				
< 1 km	108 (65.5)	96 (60.0)	Ref	Ref
1–5 km	41 (24.8)	37 (23.1)	0.99 (0.58–1.68)	0.93 (0.54–1.60)
> 5 km	16 (9.7)	27 (16.9)	0.53 (0.59–1.68)	0.50 (0.54–1.60)
Don’t know	1 (0.6)	0 (0.0)	–	-^**a**^

^a^Dropped because of a cell has < 5 observations. Bold face = statistically significant results

^b^Adjustment conducted for variables on the table

**Table 6:** Knowledge of cervical cancer symptoms and socio-demographic correlates

Socio-demographic variables	Knowledgeable on cervical cancer symptoms		Odds ratios (OR)	
	Yes N (%)	No N (%)	Crude OR (95% CI)	Adjusted OR^a^ (95% CI)
**Age (Years),** ***N*** **= 815**				
18–29	209 (51.1)	224 (55.2)	Ref	Ref
30–44	115 (38.4)	136 (33.5)	1.24 (0.92–1.7)	1.27 (0.92–1.73)
45–60	41(10.5)	46 (11.3)	1.00 (0.63–1.58)	1.07 (0.66–1.73)
**Marital status,** ***N*** **= 815**				
Married/Living together	278 (68.0)	275 (67.6)	Ref	Ref
Separated/Divorced	35 (8.6)	25 (6.2)	1.38 (0.81–2.38)	1.47 (0.83–2.62)
Single	39 (9.5)	68 (16.8)	**0.58 (0.39–0.89)**	**0.59 (0.38–0.94)**
Widowed	57 (13.9)	38 (9.4)	1.48 (0.95–2.31)	1.56 (0.96–2.55)
**Level of education,** ***N*** **= 815**				
None	111 (27.1)	84 (20.7)	Ref	Ref
Primary	246 (60.2)	278 (68.5)	0.67 (0.48–0.93)	0.73 (0.51–1.05)
Secondary	49 (12.0)	39 (9.6)	0.95 (0.57–1.57)	1.22 (0.70–2.12)
Tertiary	3 (0.7)	5 (1.2)	0.45 (0.1 1–1.95)	0.42 (0.09–2.00)
**Tribe,** ***N*** **= 815**				
Acoli	339 (97.6)	386 (95.1)	Ref	Ref
Others	10 (2.4)	20 (4.9)	0.48 (0.22–1.05)	**0.44 (0.19–0.99)**
**Employment,** ***N*** **= 815**				
No	372 (91.0)	387 (95.3)	Ref	Ref
Yes	37 (9.0)	19 (4.7)	**2.03 (1.14–3.58)**	**2.07 (1.12–3.84)**
**Distance to the nearest HF in Sudan,** ***N*** **= 815**				
< 1 km	293 (71.6)	142 (40.2)	Ref	Ref
1–5 km	85(20.8)	166 (47.0)	**0.38 (0.28–0.52)**	**0.36 (0.26–0.49)**
> 5 km	29 (7.1)	32 (9.1)	**0.51 (0.31–0.85)**	**0.49 (0.29–0.84)**
Don’t know	2 (0.5)	13 (3.7)	–	-^a^

^a^Dropped because of a cell has < 5 observations. Bold face = statistically significant results

^b^Adjustment conducted for variables on the table

## Discussion

This study provides insights into the knowledge of refugee women from South Sudan now living in Uganda on cervical cancer risk factors and symptoms. Results from this study could inform targeted interventions and health services provision back in South Sudan, having identified the target women groups from the settlement. We found that the majority of the refugee women (60%) had not heard of cervical cancer before this study. Of the women who had heard, the majority could recognize most of the cervical cancer risk factors. HPV infections, multiple male sexual partners (> 3), and early sexual debut were well recognized as risk factors for cervical cancer. However, less than half of participants recognized smoking cigarettes (38%) as a risk factor. Recogntion of risk factors was only associated with being formally employed. All participants were assessed for recognition of cervical cancer symptoms; recognition was generally low among the women: Persistent lower abdominal pain (65%) was the most recognized cervical cancer symptom, followed by persistent smelly vaginal discharge (63%) and vaginal bleeding after menopause (60%). Recognition of cervical cancer symptoms was statistically significantly associated with being married, formally employed and residing within 1 kilo meter of the the nearest health facility back in South Sudan.

Our findings are similar to results of a study from a neighbouring host community; recognition of smoking and delivery of three or more children as risk factors for cervical cancer were also low in the neighbouring host community of Gulu [33]. Other studies in East [34, 35] and South Africa [36] have similarly reported low recognition or reporting of cigarrette smoking and grand multiparity as risk factors for cervical cancer. In the Isiolo and Tharaka Nithi counties of Kenya, only 17.5% (36/360) of women recognized multipairty as risk factor for cervical cancer [35]. Studies in the Middle East have also shown low levels of awareness of cancer risk factors and symptoms among refugee women [37, 38]. While recognition of HPV was high in this study, only 32.3% of refugees in Italy recognized the HPV as a causative agent of cervical cancer [39]. Refugees in Australia, most of whom were from Sudan (25.0%, *n* = 36) and Liberia (31.3%, *n* = 45), had significantly lower levels of knowledge of cervical cancer compared to other non-refugee immigrant women [20]. Poor recognition of key cervical cancer risk factors begs for educational interventions to increase women’s awareness in order to promote risk reduction behaviours and reduce incidence of cervical cancer in the low- and middle-income countries especially among the refugees. On the otherhand, appropriate public awareness messages including on promotion of HPV vaccination could be woven within and anchored on the risk factors that participants have correctly recognized as entry points to increase acceptability of risk reduction interventions.

In this study, we found that only about half of the participants could correctly recognize cervical cancer symptoms. Our result is similar to findings from a number of studies in Sub-Saharan Africa that show low awareness of symptoms of cervical cancer in the general population [40, 41]. The relatively low level of recognition (highest 65%) may not be high enough to trigger the appropriate response when a woman in this settlement encounters such a symptom in real life. Studies in the low- and middile-income countrieas have shown that poor recognition of symptoms of cervical cancer is associated with attribution of symptoms to other mundane bodily changes or sexually transmitted infections and subsequently delay in health-seeking and advanced stage cancers at diagnoses [42–44]. There are limited studies on cancer symptoms awareness and cancers generally among the refugee population especially in Africa [45]. A recent review showed that in the whole of Africa, there were only two studies on cancers conducted among refugees in Congo Brazzaville [46]. Studies in Africa on refugees have focused mainly on reproductive health, malnutrition and communicable diseases especially epidemics [23, 47, 48]. In the Middle East, studies have shown that cancers among refugees are often paid less attention and refugees with cancers experience several barriers to care including poor access to specialized cancer treatment facilities and specialists [49–53]. International humanitarian organizations and health planners focus on communicable diseases and epidemics with high fatality including cholera, malaria and malnutritions [54]. Yet cancer diagnoses among refugees present a major health burden on the health systems of the host countries [22]. Findings from this pioneer study suggest the need for public health interventions aimed at increasing awareness of cervical cancer symptoms among the refugee women. Until the international communities refocus their attentions to cancers among refugees, this sub population will remain neglected and experience inequity in access to appropriate healthcare, and poorer survival from cancers.

### Strengths and limitations of the study

Majority of the participants in this study spoke Acoli, the local language of the host community. The questionnaire used was in Acoli, and the RAs were also ethnic Acoli; therefore, the questions could have been well understood. The findings perhaps reflect the true cervical cancer knowledge among women in the settlement. However, the study has some limitations. First, although the knowledge assessment approach applied in this research has been validated in the AWACAN tool referred to earlier, there is possibility that prompted items addressing recognition of symptoms/risks factors may overestimate awareness while unprompted questions on cancer awareness may underestimate awareness. Second, this was a cross sectional study that depended on self-report by the participants. Recall bias and social desirability bias could affect their responses. Third, the settlemnet commandants were strict on access to available health services and limited our access to what was in our protocol. We therefore can not tease out the contributions of health programmes conducted in the settlement to the knowledge enhancement of the participants. Finally, generalization of findings from this study to inform health services intervention in South Sudan or other refugee settings need to take into considerations knowledge and behavioural changes that could have been introduced by different actors during encampment in this settlement.

## Conclusions

More than half of the refugee women from South Sudan had not heard of cervical cancer before this study. These women are therefore unlikely to undertake preventive measures including cervical screening and or seek appropriate care when symptomatic. There is need for deliberate public awareness interventions to improve recognition of cervical cancer risk factors and symptoms, and to increase awareness of the benefits of treatment when cervical cancer is detected in early stages. Single women and women who lived farther than one kilo meter from the nearest health facility in South Sudan are a particular target group for health education interventions regarding cervical risk factors and symptoms.

## Data Availability

The datasets used and/or analyzed during the current study are available from the corresponding author on reasonable request.
